# Endovascular Treatment of Vascular Injuries in the Craniocervical Region With a Graft Stent: A Single-Center Experience

**DOI:** 10.7759/cureus.47323

**Published:** 2023-10-19

**Authors:** Anıl Tanburoglu, Cagatay Andic

**Affiliations:** 1 Neurology, Başkent University, Adana, TUR; 2 Interventional Radiology, Faculty of Medicine, Baskent University, Adana, TUR

**Keywords:** graft stent, trauma, injury, vertebral artery, carotid artery

## Abstract

Aim: We aimed to evaluate the efficacy and safety of graft stent implantation in the endovascular treatment of vascular injuries in the craniocervical area.

Materials and methods: This study was carried out through the retrospective screening of eight (two females and six males) patients' records. Patients who used graft stents as an endovascular method were included in the study. The mean age of the patients was 43.6 years (with a range of 15-69 years). Due to different mechanisms, the patients had vascular injuries in the craniocervical region, and graft stent implantation was performed between 2010 and 2022. We evaluated patient demographics, admission symptoms, trauma mechanisms, angiographic findings, treatment modalities and materials, patient outcomes, and follow-up periods.

Results: Due to iatrogenesis for four patients, penetrating trauma for three patients, and blunt trauma for one patient, injuries were present in the right internal carotid artery {ICA} (n=1), left ICA (n=1), left common carotid artery {CCA} (n=3), right CCA (n=1), right vertebral artery (n=1), and left vertebral artery (n=1). Angiographically, pseudoaneurysm was detected in seven patients, and vascular rupture was detected in one patient using contrast agent extravasation. All patients who underwent the endovascular method had technical success. Since bleeding from the external carotid artery branches was seen in two patients, these branches were embolized with coils. No radiologically or neurologically pathological findings were recorded during the follow-up period (with a range of one week to 12 years).

Conclusion: Graft stent use in the endovascular treatment of craniocervical vascular injuries is an operable, safe, and promising option, especially in patients with pseudoaneurysms and active bleeding.

## Introduction

Craniocervical region vascular injuries resulting from trauma are the primary cause of mortality and morbidity worldwide [1]. Furthermore, these injuries may be responsible for up to 20% of trauma death regardless of the mechanism [2-4]. Direct or indirect injury to the craniocervical region may result from an accident, tumor abrasion, or even spontaneously arise [5]. Although blows are the most common cause of head injuries, sudden body deceleration is also a factor [6].

Vascular injuries can occur in blunt trauma, penetrant trauma, or iatrogenesis [7]. Penetrating vascular injuries are estimated to account for 5-15% of traumas [8]. The most common types of penetrating vascular injuries are skull fractures, gunshot wounds, stabbings, as well as direct damage caused by a penetrating material [3].

Cerebrovascular injuries from blunt trauma are less common, with a 1.0-1.6% prevalence in the traumatic patient population [9]. It is usually the result of motor vehicle accidents (61%) [10]. Iatrogenic injuries resulting from different diagnostic or therapeutic procedures are uncommon but can potentially be fatal complications [2,11].

Active bleeding from carotid or vertebral lacerations can lead to a high risk of stroke and death. The risk remains significant even with aggressive treatment, such as surgical ligation or endovascular embolization of the vessel. The surgical approach to the skull base carotid and vertebral artery is problematic. Therefore, interventional endovascular treatment approaches can significantly support the trauma team [1].

Pseudoaneurysms result from arterial bleeding into the wall of an injured vessel. They may present as a focally enlarged dissection surrounded by the adventitia or retention of leakage outside the vessel wall by a layer of a clot. Conservative therapy is rarely effective in the treatment of traumatic pseudoaneurysms. The surgical approach has been the classical method for many years. However, surgical repair is unsuitable for treating traumatic carotid pseudoaneurysms due to the anatomical difficulties that limit the surgical approach and procedure-related stroke and mortality rates of up to 9% [3].

As a result, the use of endovascular techniques has shown promise in recent years and has established itself as a treatment strategy. However, although using graft stents in endovascular therapy has reported promising rapid results in a few small-scale case series, little is known about long-term outcomes [4,5].

This study aimed to evaluate the efficacy and safety of graft stent implantation in the endovascular treatment of vascular injuries in the craniocervical region. It also aimed to report whether it is a safe and feasible option for its early and long-term results.

## Materials and methods

Patient characteristics

This study was approved by the Baskent University Institutional Review Board (#KA21/478). This study was carried out by a retrospective screening of patients with vascular injury with different mechanisms and graft stent implantation in the craniocervical region between 2010 and 2022. This study involved eight patients (two females and six males) with a mean age of 43.6 (range: 15-69) who suffered a vascular injury as a result of iatrogenic, blunt, or penetrating trauma and were treated with graft stent implantation. Three operators evaluated angiographic findings. They all had at least five years of experience in interventional radiology or neurology. They performed this evaluation by scanning the hospital's registry system, which contained information on the selected patients. Carotid occlusion, stenosis, pseudoaneurysm, dissection, carotid-cavernous fistula, and contrast extravasation were recorded. The patient's demographic information, admission symptoms, trauma mechanism, angiographic findings, treatment method and materials used in treatment, patient results, and follow-up periods were obtained from the electronic record system of our hospital.

Four of these patients had iatrogenic injuries, and two of them could not be controlled with massive oral and anterior or posterior nasal packing or by compressing the local damaged area. These patients are hemodynamically unstable. There was a patient diagnosed with a rapidly developing hematoma in the right half of the neck accompanied by airway compression symptoms following a carotid artery rupture during an internal jugular vein (IJV) catheterization. Another iatrogenic injury was detected on computed tomography angiography (CTA), which was taken after a sudden onset of hematoma in the posterior part of the right neck and associated compression symptoms after facet joint injection.

Blunt trauma was accompanied in one patient, and vascular injury in the craniocervical region due to penetrating trauma in three patients. Blunt trauma was due to traffic accidents, and penetrating injuries were injuries from gunshot wounds or glass cuts. The blunt trauma had a concomitant LeFort III maxillofacial fracture. In addition, the patient, who was noticed on CT scans and had a clinically stable appearance, had pseudoaneurysmatic excess filling in the right internal carotid artery (ICA) petrous segment. One of the patients with penetrating trauma was pregnant at 24 weeks. The patient sought emergency medical attention due to the sinking of the glass piece, which caused a deep incision on the left half of the neck. After a color Doppler ultrasonography (CDUS) examination, the patient was consulted. The examination results showed excessive pseudoaneurysmatic filling in the left common carotid artery (CCA). The other two patients with penetrating injuries were consulted after CTA was taken and admitted to the emergency department due to gunshot wounds. One of the patients had a left vertebral artery injury, and the other had a left ICA injury. Clinically, both patients had no neurological deficits, but both patients had unstable vital findings.

All eight patients were quickly evaluated after consultation with the interventional radiology unit, and they were taken to the angiography suite, and treatment was started. Almost all patients, including those with hemodynamic instability, were treated quickly by an experienced angiography team. The angiography suite is always ready for unstable hemodynamic patients or patients requiring urgent intervention.

Interventional management

The patients were seen in an emergency setting, and just before stenting, they were given 100 mg of aspirin and 300 mg of clopidogrel via a nasogastric tube. In addition, Aggrastat (glycoprotein IIa/IIIb receptor blocker) was prepared against acute stent thrombosis. However, acute stent thrombosis was not observed in any of the patients. For this reason, none of the patients needed to use Aggrastat. The transfemoral approach was preferred during the procedure to obtain a complete neuroangiogram of the carotid and supra-aortic arteries and their branches, including the vertebral arteries.

We placed a 10F vascular sheath in the main femoral artery in patients who decided to place a graft stent. The standard heparin dose (approximately 70-100 U/kg) in carotid or other supra-aortic stent angioplasty applications was injected IV to keep the activated coagulation time (ACT) above 250 seconds. Patients with unstable vital signs were given IV heparin just before stent-graft placement.

**Figure 1:**
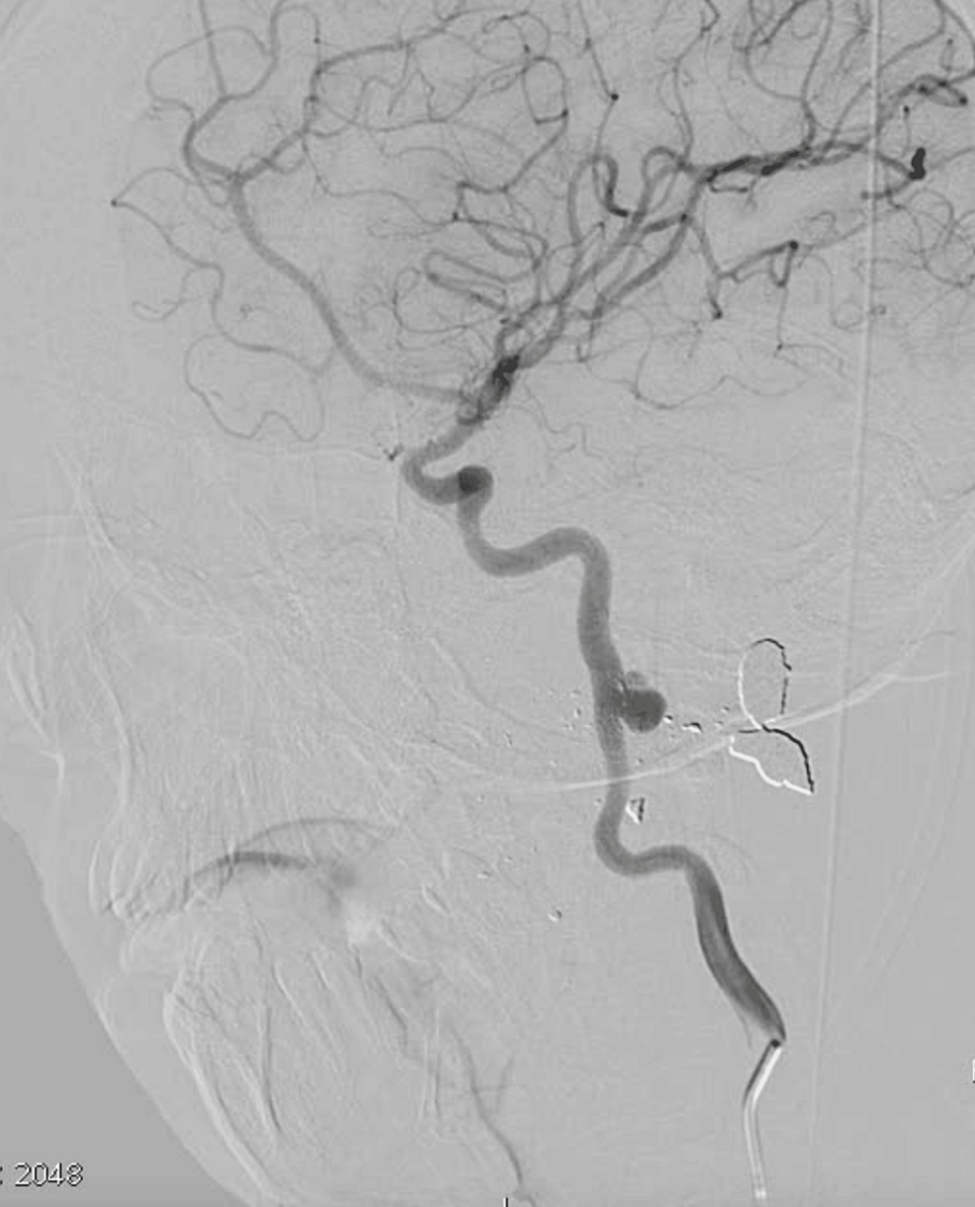
DSA detected a wide-necked pseudoaneurysm in the left ICA cervical segment. Fifteen-year-old male patient after a gunshot wound. DSA: digital subtraction angiography; ICA: internal carotid artery

**Figure 2:**
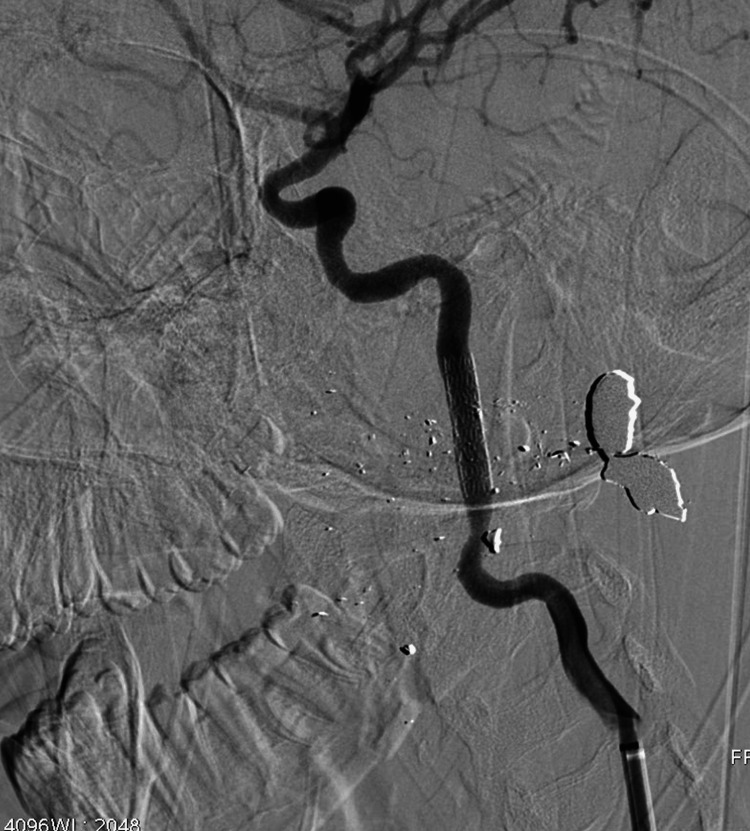
Successful treatment is followed after graft stent implantation. Fifteen-year-old male patient after a gunshot wound.

**Figure 3:**
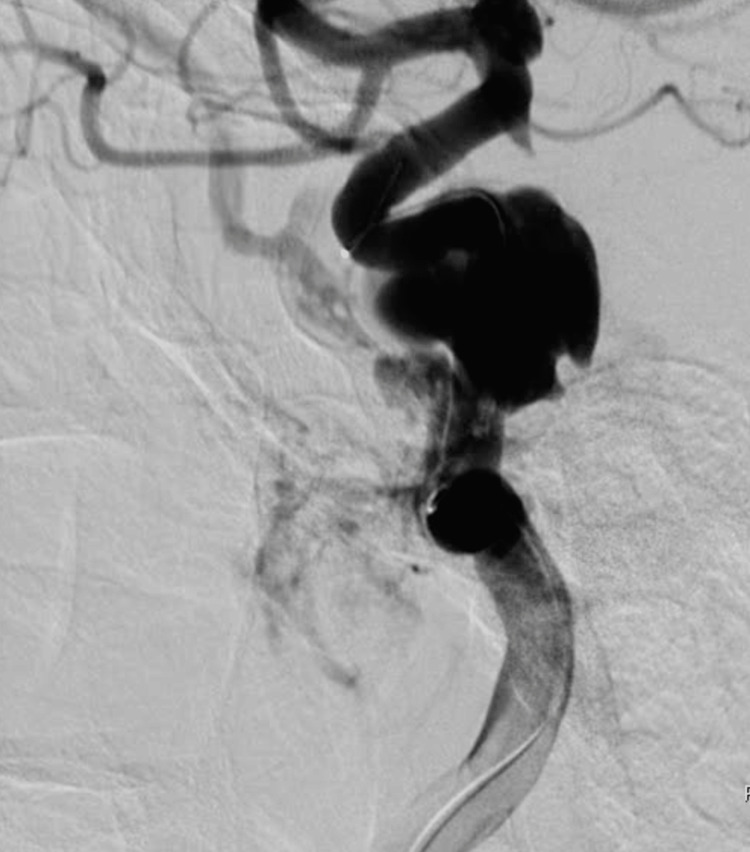
Magnify images in lateral projection at DSA. Wall defect and pseudoaneurysm from petrous segment to cavernous segment (In the left distal ICA lateral wall). In a 53-year-old female maxillofacial trauma after an in-car traffic accident. DSA: digital subtraction angiography; ICA: internal carotid artery

**Figure 4:**
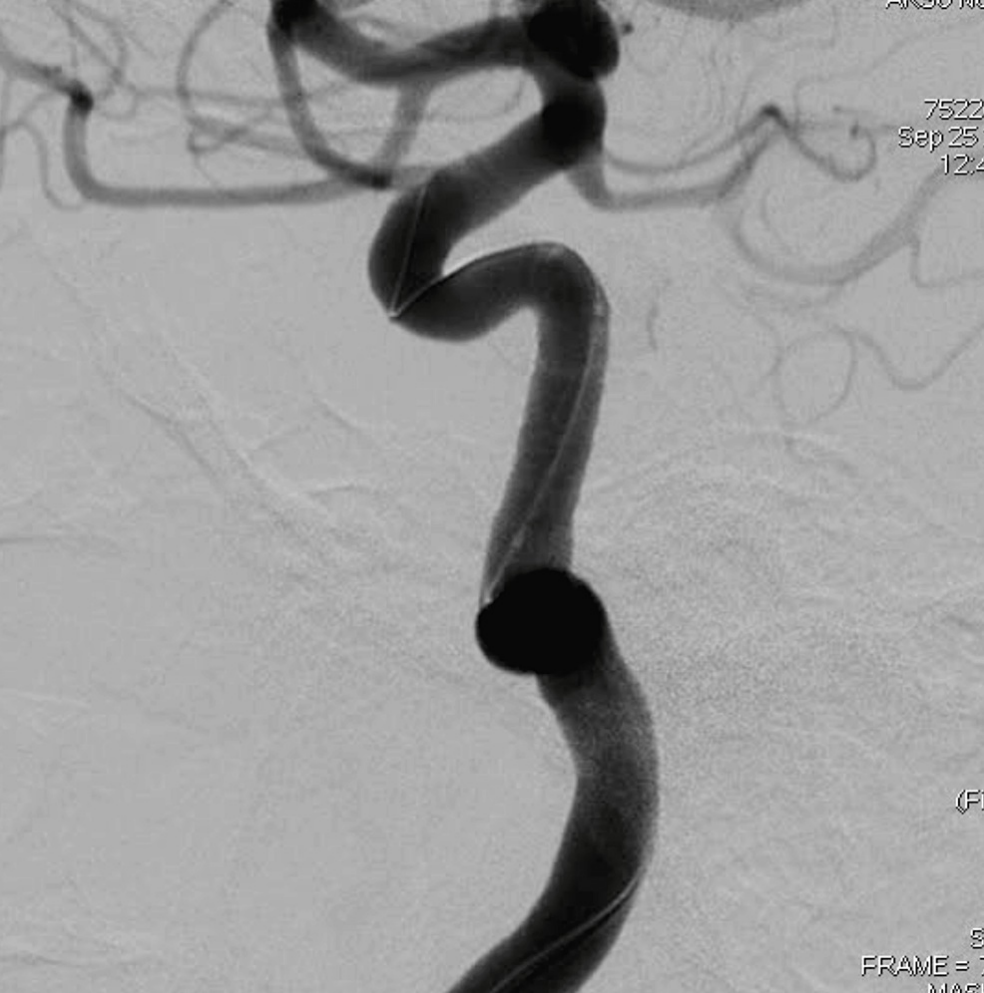
DSA images after graft stenting. The graft stent repaired the vascular defect. In a 53-year-old female maxillofacial trauma after an in-car traffic accident. DSA: digital subtraction angiography

Selective images were obtained by advancing a 5F diagnostic catheter (vertebral or Simmons 2; Bloomington, IN: Cook Medical Inc.) into the proximal segment of the CCA or the vertebral artery origin. A stent graft was selected in width and length suitable for the diameter of the diseased segment to cover the entire damaged segment by performing magnified angiographies. Our covered stent is a self or balloon-expandable nitinol stent coated with polytetrafluoroethylene (PTFE). Different stents were preferred for the anatomical localization of the pathology. The preferred graft stents in the vertebral artery and distal ICA are Graftmaster (Santa Clara, CA: Abbott Vascular Inc) and PK Papyrus (Büloch, Switzerland: Biotronic AG). In addition, the preferred stents in the cervical segment of the carotid artery are LifeStream (Wexford, Ireland: Bard Peripheral Vascular, Inc.) and Fluency Plus (Karlsruhe, Germany: Bard GmbH). Figures 1-4 depict the post-management angiograms.

Analysis

We analyzed initial clinical and angiographic results after graft stent placement. In addition, this study examined recurrence rates of bleeding, neurological problems, presence of infection, surgical or endovascular treatment management, and clinical follow-up. The results were rated as "good" in patients who experienced technical success with clinical early bleeding control and no procedure-related complications.

## Results

In total, there were two females and six males between the ages of 15 and 69 years. Five vascular injuries occurred in the extracranial ICA, two in the intracranial ICA, and two in the vertebral artery (Table 1).

**Table 1:** Patient population in the study and treatment modality, follow-up period, and results. M: male; F: female; ICA: internal carotid artery; ECA: external carotid artery; CCA: common carotid artery; IJV: internal jugular vein; V2: vertebral artery segment 2; V3: vertebral artery segment 3

Case	Age/gender	Cause	Symptom of application	Treated vessel	Angiographic findings	Treatment	Diameter of the stent used in treatment	Result	Follow-up period
1	53/F	Blunt trauma	Traffic accident, maxillofacial trauma (LeFort III, facial fracture)	Right ICA	Defective appearance and pseudoaneurysm sac on the ICA lateral wall in the area extending from the right ICA petrous segment to the cavernous segment	Graft stent implantation	4.8×16 mm	Good	6 years
2	15/M	Penetrant trauma	There is active bleeding from the entrance hole in the left cheek following the shooting, but there is no evidence of neurological damage	Left ICA	Wide-neck pseudoaneurysm sac in the left ICA cervical segment	Graft stent implantation	6×58 mm	Good	4 years
3	30/M	Iatrogenic injury	Hematoma on the neck, after facet joint injection	Right vertebral artery	Pseudoaneurysm sac in the V2-V3 segment of the right vertebral artery	Graft stent implantation	4×20 mm	Good	2 years
4	63/M	Iatrogenic injury	While inserting the catheter from the right IJV. Large hematoma causing pressure on the right side of the neck	Right CCA	Pseudoaneurysm sac in the right CCA	Graft stent implantation	9×38 mm	Good	1 year
5	66/M	Iatrogenic injury	Larynx cancer, intraoperative bleeding	Left CCA	Severe contrast extravasation and rupture in the left CCA after the external tampons were removed+rupture of the left ECA ramuscules were observed	Graft stent implantation to the left CCA+coil embolization for bleeding in the left ECA ramuscules	9×80 mm	Good	5 weeks
6	69/M	Iatrogenic injury	Larynx cancer, intraoperative bleeding	Left CCA	Pseudoaneurysm	Successful bleeding control was achieved with the ECA and graft stent	8×60 mm	Good	1 week
7	24/F	Penetrant trauma	The neck has been injured by a piece of glass; there is no neurological deficit. Hematoma on the left side of the neck. An RDUS examination revealed a pseudoaneurysm in the left CCA. Patient who is 24 weeks pregnant	Left CCA	Pseudoaneurysm sac with a diameter of 2 cm was observed in the superior part of the left CCA	Graft stent implantation was performed	8×30 mm	Good	12 years
8	29/M	Penetrant trauma	Neck injury caused by a gunshot. Neurological symptoms are not present. Left side of the neck bleeding from the entrance hole	Left vertebral artery	3×3 mm pseudoaneurysm sac in the V2 segment of the left vertebral artery	Graft stent implantation was performed	5×16 mm	Good	7 years

Angiographically, seven patients had pseudoaneurysms. In one patient, severe contrast extravasation was observed when removing the detected compression sponges. In addition, external carotid artery (ECA) branches in two patients accompanied by contrast extravasation.

Technical success was achieved in all patients (100%). No technical difficulties were encountered during the placement of any of the stents. No complications were observed during and immediately after the procedure. No patient had recurrent bleeding, infection, neurologic problem, or need for surgery during the post-procedure follow-up. Clinically, bleeding was stopped in the early period after the graft stent and hemodynamic findings were stable. Two patients with oropharyngeal cancer died of metabolic and cardiac causes (one and five weeks post-procedure). These patients are out of follow-up. As for the rest of the patients, they had various follow-up periods ranging from one to 12 years. No potential complications (including death, stroke, vascular injury, stent thrombosis, endoleak, and intrastent stenosis) were recorded during follow-up. The results were evaluated as "good" in all patients due to technical success, early bleeding control, and no procedure-related complications.

## Discussion

This study presents the short-term and long-term results of graft stent use in eight patients with life-threatening vascular trauma in the craniocervical region. This procedure may be considered as an alternative for these types of injuries, which are difficult to treat surgically.

Blood vessels are often affected after neck trauma. The most commonly injured zone is zone II (cricoid cartilage to the angle of the mandible (47%), followed by zone III (from the angle of the mandible to the skull base) (19%) and zone I (from the sternal notch to the cricoid cartilage) (18%) [6]. In our study, one of our patients had a penetrating injury with a gunshot wound. There was another case in which a glass fragment was found in zone II of the neck, the most commonly affected area reported in the literature. Two of our patients suffered blunt trauma and another sustained a penetrating injury. Zone III was the location of these injuries.

The mortality rate is 80% in patients with a late diagnosis of craniocervical injuries [7]. Therefore, screening criteria based on signs and symptoms and risk factors have been proposed for these patients [8,10]. DSA remains the gold standard for blunt neck injuries. However, CTA, CDUS, and magnetic resonance angiography are the recommended non-invasive imaging modalities [6]. There have been no controlled randomized studies for optimal management in blunt carotid artery trauma patients. The primary treatment modalities are anticoagulation/antiaggregant therapy, open surgery, and endovascular therapy [6]. Biffl et al. proposed an angiographic scale including various degrees of injury in blunt carotid artery injuries [9]. One of our patients had a blunt carotid artery injury, one of the most frequently injured areas cited in the literature, at the skull base level and accompanying LeFort III facial fractures. No neurological findings were observed except clinically severe headache. Therefore, CTA was performed for screening purposes in this patient. However, a pseudoaneurysm sac was detected in a difficult-to-access area of the right ICA petrous segment. As a result, the decision was made to perform the endovascular treatment to address the issue.

Iatrogenic vascular injuries in the craniocervical region are rarely seen during diagnostic or therapeutic procedures. However, they cause high mortality and morbidity rates. As a result, preparation is essential when treating iatrogenic injuries. In addition to perioperative bleeding and strokes, death can also occur as a result of these complications [11,12]. In our study, four patients had iatrogenic injuries. The most important feature of these patients is the absence of neurological symptoms. However, in all of them, bleeding and hematoma were detected in the perioperative period.

In the literature on craniocervical zone injuries in endovascular treatment, parent artery occlusion (with coil or detachable balloon, vascular plug, or liquid embolic agent) [11]; self-expanding stent and coiling [13,14]; stent-assisted coiling [11,14,15]; balloon-assisted coiling [16]; graft stent implantation [14,17,18]; self-expanding stents [14,17]; and closure of the aneurysm with primary coiling have been reported [14,18]. However, the natural history and appropriate management of carotid artery injuries in the craniocervical zone have not been fully defined. The primary treatment for patients with carotid artery trauma is anticoagulants or antithrombotic agents. These medications are considered the first line of defense in preventing further complications. Endovascular techniques are essential in selected cases, especially in pseudoaneurysms [14,19].

Assadian et al. reported that one patient had a transient ischemic attack during the procedure [20]. However, there were no late complications up to six months in a series of six patients with internal carotid artery dissection (spontaneous or post-traumatic) in whom graft stenting was applied. Maras et al. conducted a study involving 20 patients with penetrating craniocervical injuries and skull base fractures [5]. The patients were treated with a graft stent for extracranial internal carotid artery pseudoaneurysms. During the follow-up period, an in-stent occlusion rate of 15% was reported in the study. They interpreted this rate as acceptable due to the complexity of the injuries.

According to Joo et al., 10 patients were treated, nine with blunt carotid artery injuries and one with a penetrating carotid artery injury [21]. They treated a pseudoaneurysm with graft stent implantation in a patient with a penetrating carotid injury. The follow-up results of this study reported that endovascular treatment of carotid injury was safe and feasible. In most of the literature, endovascular therapies are often preferred for treating pseudoaneurysms that are difficult to locate and remove surgically [22]. McNeil et al. reported successfully treating the carotid artery's pseudoaneurysm at the skull's base in zone III after a bullet exposure with a graft stent [22]. Mei et al., in their study of 11 patients on endovascular treatment of high-grade traumatic vertebral artery injuries, detected pseudoaneurysm in 10 patients and arteriovenous fistula in one patient [4]. One of the patients with pseudoaneurysm was treated with a graft stent. The nine other patients were treated with stent-assisted coil embolization. In the follow-up of these 10 patients, no stenosis or aneurysm requiring treatment was found in the control examinations.

As a result of these findings, the authors concluded that endovascular treatment is essential in preventing permanent neurological damage and surgical risks in high-grade traumatic vertebral artery injuries. Aydin et al. conducted a study on the endovascular treatment of iatrogenic craniocervical vascular injuries [11]. In the study, they treated a patient with a carotid-cavernous fistula using a graft stent among the 21 patients. Although the follow-up period was noted as one month for this patient, the result was interpreted as good, despite the short follow-up period. In addition, 15 patients were treated with parent artery occlusion in this study. Stent-assisted coiling in one patient and primary coiling in another closed their aneurysms. The other two patients were followed up with a conservative approach. They interpreted endovascular therapy as a good option with low mortality and morbidity rates. This is especially true for rare iatrogenic vascular craniocervical region injuries. In our study, the graft stent was successfully inserted in all patients, providing bleeding control and treatment of the pseudoaneurysm. Furthermore, no neurological deficit was detected in our patients during the procedure or their post-operative follow-up. Our results confirm and support the literature.

Conservative treatment rarely effectively treats traumatic carotid pseudoaneurysms [3,23]. Anticoagulant treatment is typically recommended for traumatic dissections, but it is not expected to promote spontaneous recovery in cases of pseudoaneurysms. Therefore, it is contraindicated in pseudoaneurysms after a severe injury and multiple trauma. The treatment approach involved commonly used surgical methods such as aneurysm clipping, resection, end-to-end anastomosis, resection and interposition graft, extracranial-intracranial bypass, and vessel ligation. However, procedure-related stroke and mortality rates of up to 9% have been reported in surgical methods. Graft stents are a novel reconstructive treatment strategy in minimally invasive endovascular therapy. It creates a direct physical barrier and abruptly closes the leak. It is the most effective treatment method for maintaining normal physiology. However, clinical cases of the use of graft stents in traumatic pseudoaneurysms are few, most are based on case reports, and there is insufficient information on long-term outcomes. A study by Wang et al. on long-term results reported that no ischemic stroke was recorded in the mean clinical follow-up of 44±16 months [3]. The pseudoaneurysm was closed, and the stents were open in the 39±16 month imaging follow-up. Maras et al. in their study reported that they followed only two cases for two years [5]. The most prolonged follow-up period was 36 months in the study by Wang et al. [2]. In this study, the patient with the most prolonged follow-up period was followed for 12 years (an average of 4.2 years, excluding patients excluded from early follow-up). It is one of the studies with the most extended follow-up series described in the literature.

Stents have been used in vertebral artery injuries less frequently than in carotid artery injuries, but reports of their use have gradually increased in the last decade. All patients treated using a graft stent responded well to treatment. However, no details were given about the stent types used [24]. In our study, balloon-expandable graft stents were placed in vertebral artery injuries. We rated the long-term results as excellent. The literature on traumatic penetrating carotid artery injuries or blunt vertebral artery injuries is more extensive [24-26]. Therefore, the findings of this study will contribute to developing treatment algorithms for penetrating vertebral artery injuries. The results of our study are remarkable, considering both the stent types used and the long-term results.

Limitations 

There are two main limitations of this study. The first is that the use of retrospective screening limits the findings of this study; the second limitation is the varying patient follow-up times.

## Conclusions

Vascular injuries to the craniocervical region are uncommon, but they can be life-threatening. Thus, early diagnosis and treatment are essential. Our review indicates that graft stents are a safe and promising endovascular treatment option. In particular, this applies to patients with pseudoaneurysms and active bleeding.
